# Urban tree species benchmark dataset for time series classification

**DOI:** 10.1016/j.dib.2025.111777

**Published:** 2025-06-20

**Authors:** Clément Bressant, Romain Wenger, David Michéa, Anne Puissant

**Affiliations:** a LIVE UMR 7362 CNRS, University of Strasbourg, 3 rue de l’Argonne, Strasbourg, 67000, France; b EOST UAR 830 CNRS, University of Strasbourg, 5 Rue René Descartes, Strasbourg, 67000, France; c UAR 2013 CNRS, Data Terra / THEIA, 500 rue Jean François Breton, Montpellier, 34090, France

**Keywords:** Urban trees, Sentinel-2, PlanetScope, Time series classification, Deep learning

## Abstract

Classification of urban tree species is essential for understanding their ecological functions, managing urban forests (public and private), and informing nature-based solutions for climate resilience. We present a benchmark dataset for urban tree species classification based on multi-source optical satellite image time series (SITS). The dataset provides, on the city of Strasbourg (France), surface reflectance values extracted from coregistered Sentinel-2 and PlanetScope imagery on public trees. Species labels and geolocations are derived from the city inventory *Patrimoine arboré 2022*. A total of 45,084 trees representing the 20 most common species are included. The dataset is formatted for time series classification, with surface reflectance values and consistent spatial sampling. It supports direct integration into deep learning frameworks and includes three InceptionTime-based models trained on Sentinel-2, PlanetScope and both sources through a fusion architecture (Dual-InceptionTime). Model outputs—predicted species, confidence scores, and correctness flags—are provided, along with an interactive t-SNE visualization of the latent feature space for interpretability and error analysis. This dataset offers a reproducible framework for evaluating species classification models, fusion strategies, and explainability techniques, and contributes to advancing urban vegetation monitoring using satellite image time series and deep learning.

Specifications Table

**Table:** 

Subject	Earth & Environmental Sciences
Specific subject area	Remote Sensing, Deep Learning, Urban Ecology
Type of data	GeoPackage (GPKG) and Python code (IPYNB, PY)
Data collection	Polygon-based values from coregistered L2A Sentinel-2 and L3B PlanetScope image time series (2022). The surface reflectance values are available for each public tree in the urban area, linked to point with labels from a local expert database (Patrimoine arboré 2022 - OpenDataStrasbourg). This benchmark dataset is processed in deep learning models (InceptionTime), with a fusion-based approach (Dual-InceptionTime). Python code and outputs are provided for the various model architectures, along with an interactive tool to explore classification errors and species-level confusion.
Data source location	Urban area of Strasbourg, Grand-Est, France (48.57-48.62°N, 7.72-7.80°E)
Data accessibility	The two GeoPackage files—one with raw time series values and the other with processed model outputs—are available as follows: Repository: EaSy Data Data identification number: 10.57932/c17e2153-c687-4c28-824c-2be148fb9e4d Direct URL to dataset: https://doi.org/10.57932/c17e2153-c687-4c28-824c-2be148fb9e4d Source code for the deep learning models is publicly available on GitHub: https://github.com/r-wenger/Urban-tree-classification The interactive t-SNE plot can be accessed at: https://romainwenger.fr/urban-trees/web_tsne_errors.html The primary data on tree locations are available in vector format from Ville et Eurométropole de Strasbourg, as follows: Repository name: OpenDataStrasbourg Data identifier: patrimoine_arbore Direct URL to dataset: https://data.strasbourg.eu/explore/dataset/patrimoine_arbore/

## 1 Value of the Data

• This dataset serves as a benchmark resource for the classification of urban tree species from satellite image time series, enabling reproducible comparisons across models, fusion strategies, and explainability techniques. To our knowledge, it is the first openly available dataset combining species-level tree annotations with multi-source time series (Sentinel-2 and PlanetScope) at the urban scale.
• The data include time series of surface reflectance values from both Sentinel-2 (10 bands, 10–20m, 22 images in 2022) and PlanetScope SuperDove (4 bands, 3.125m, 45 images in 2022) for each public tree. By providing preprocessed, ready-to-use data, the dataset removes the need for users to engage in computationally intensive and time-consuming image processing steps.
• The dataset is formatted for time series classification tasks, featuring temporally aligned observations, and patch-level sampling around individual tree locations, enabling seamless integration into deep learning frameworks. Suitable model codes for species classification, including InceptionTime and the fusion-based Dual-InceptionTime architecture, are also accessible.
• The outputs from the associated deep learning models are provided, including predicted species, confidence scores, and correctness flags. A t-SNE latent space embedding is also provided to support post-hoc analysis of model performance, feature separability, and classification error patterns.

## 2 Background

Urban trees provide essential ecosystem services that contribute to urban resilience and the well-being of city dwellers [1]. These services are largely determined by species-specific functional traits—structural and physiological—that influence ecological performance [2]. Yet, identifying and mapping tree species at scale remains a major challenge due to the high species diversity and the limitations of traditional inventories, which are costly, time-consuming, and often restricted to public spaces [3].

To address these limitations, remote sensing has emerged as a promising alternative. Multi-temporal satellite imagery allows the monitoring of vegetation dynamics, including phenological patterns that are informative for species discrimination [4]. However, extracting accurate species information from these rich temporal signals requires the use of robust analytical methods. Deep learning (DL) models are increasingly favored for this task, as they can automatically extract relevant features from raw time series and handle complex temporal structures [5]. Recent work [6] further supports this approach, demonstrating that multi-spectral data significantly enhance the classification of urban tree species using DL methods.

## 3 Data Description

**Fig. 1:**
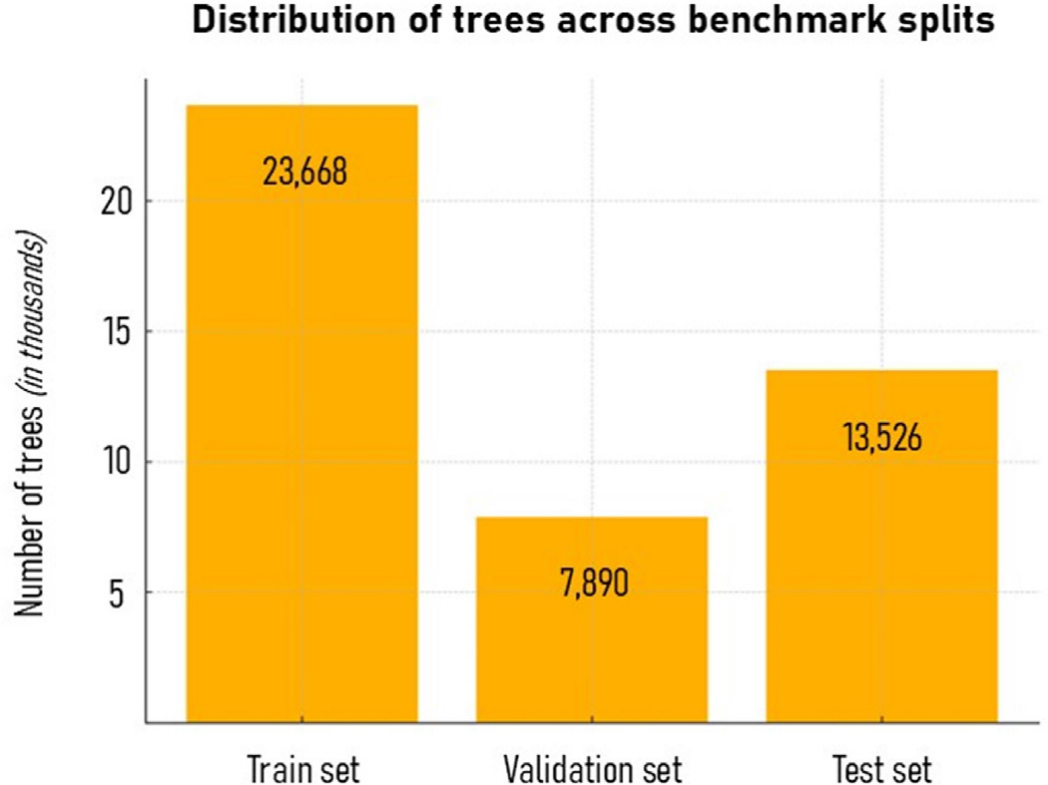
Number of trees across the train, validation, and test splits of the benchmark dataset.

We introduce a new dataset and classification framework specifically designed for urban tree species mapping using optical satellite image time series (SITS). The GeoPackage dataset (raw dataset) contains surface reflectance values acquired in 2022 from Sentinel-2 (S2) and PlanetScope (PS) satellites. Time series values are stored in Real format and structured by sensors and spectral bands. These reflectance series are spatially anchored to a city services database of public trees, which provides unique identifiers (*tree_id-num_arbre*), species labels (*true_species*), and precise geolocations across the city (from the database OpenDataStrasbourg *Patrimoine arboré 2022*). The dataset includes the 20 most common tree species, covering a total of 45,084 individual trees (split into training, validation, and test sets; see Fig. 1). Two examples of its applications can be found in [6,7].

The dataset was implemented in three InceptionTime-based models [8], aiming to classify 20 tree species from Sentinel-2 and PlanetScope SITS. Two models were trained separately on each sensor (InceptionTime-20-S2 and InceptionTime-20-PS), and a third model was developed using a fusion-based architecture (Dual-InceptionTime) to jointly leverage both data sources. A GeoPackage file is provided (processed dataset), containing model predictions for each tree of the test set (13,526 trees), including predicted species (*predicted_species*) and a binary classification flag (1: correct prediction, 0: incorrect prediction; *prediction_flag*). Results are organized by model (see the next section): (i) Dual-InceptionTime-20-S2/PS (*DualIT*), (ii) InceptionTime-20-S2 (*ITS2*), and (iii) InceptionTime-20-PS (*ITPS*). The corresponding Python code for model implementation and inference is also available in a GitHub repository.

The proposed framework enables automated tree species classification and supports post-hoc analysis of classification errors. To facilitate interpretation, we also include an interactive (web) t-SNE visualization to explore model behavior and species-level confusion.

## 4 Experimental Design, Materials and Methods

### 4.1 Surface reflectance data

Accurate urban tree species classification requires harmonized, high-quality reflectance data from satellite sensors that offer suitable spatial and spectral resolutions. Fig. 3 illustrates the acquisition workflow of the (raw) benchmark dataset.

#### 4.1.1 City tree dataset

The original data underlying this benchmark come from the *Patrimoine arboré 2022* municipal database, in which each tree is geolocated as a vector point. This dataset includes all trees individually inventoried and monitored by the departments of the *Eurometropole de Strasbourg*, regardless of the managing service. It excludes forested areas, woodland clusters, and trees located on private property.

The database is updated monthly; the version used here corresponds to the March 2022 update. After extracting the 20 most represented species, the benchmark dataset contains 45,084 trees.

#### 4.1.2 Satellite image time series

**Fig. 2:**
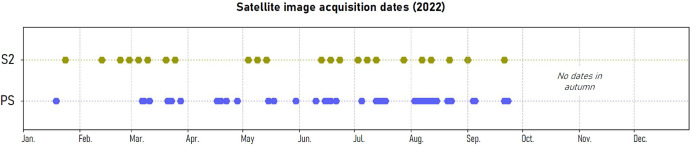
Satellite image acquisition dates for Sentinel-2 (22 dates) and PlanetScope (45 dates), for the year 2022.

Sentinel-2 imagery was obtained from the Copernicus Open Access Hub and includes data from both Sentinel-2A and 2B platforms. We selected Level-2A (L2A) surface reflectance products, which are corrected geometrically, radiometrically, and atmospherically. These multi-spectral data offer a frequency of five days revisiting, allowing 22 images (32UMU tile) to be gathered (Fig. 2). Among the 13 available spectral bands, 10 bands with a spatial resolution of 20 m or finer were retained, as coarser resolutions are unsuitable for capturing the spatial extent of individual urban trees. No resampling is necessary due to the selection of training trees taking into account the risk of autocorrelation.

**Fig. 3:**
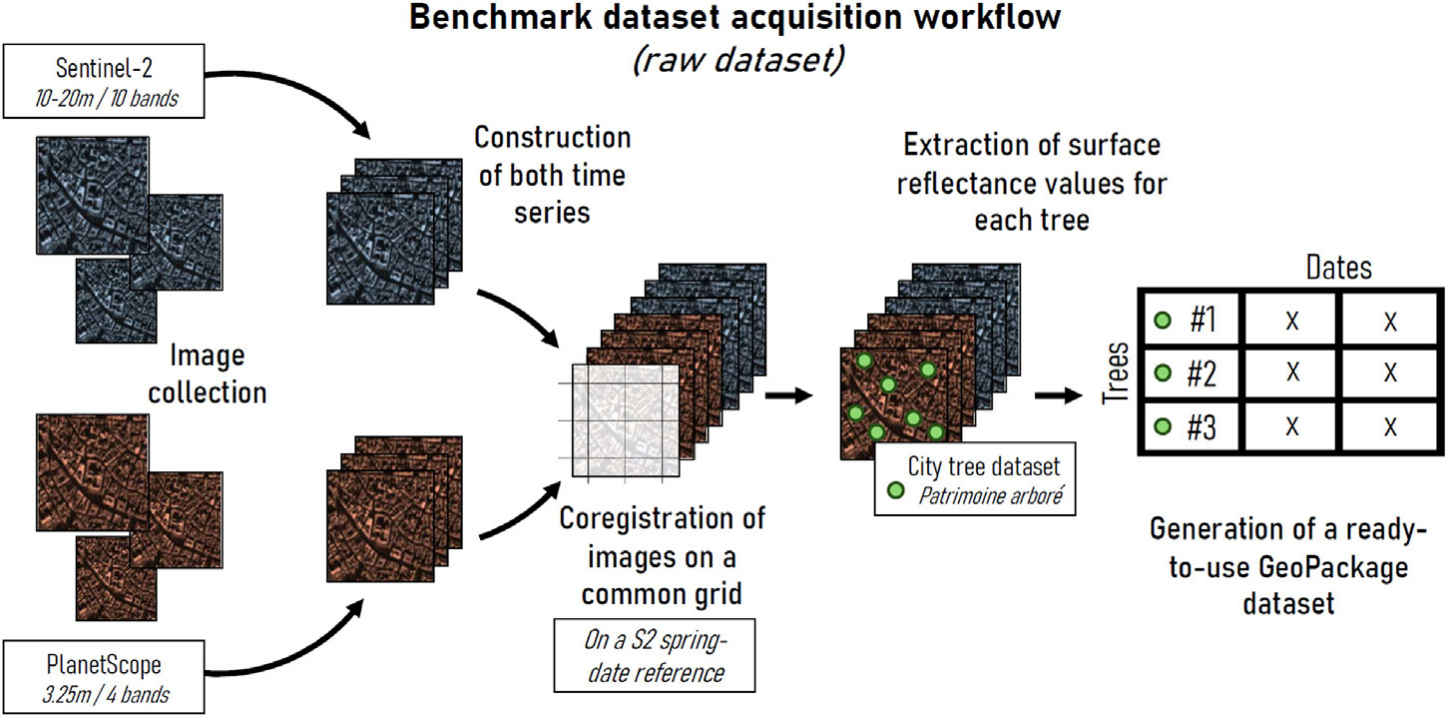
Workflow for generating the benchmark dataset from satellite image time series. The resulting GeoPackage file is used as input for deep learning models.

PlanetScope data were retrieved through Planet’s data delivery API, specifically selecting Level-3B (L3B) Ortho surface reflectance (*analytic_sr* assets) products from the SuperDove (PSB.SD) satellites [9]. These advanced sensors provide daily imagery in four spectral bands (blue, green, red, near-infrared) at a spatial resolution of 3.125 meters. In total, 45 corrected Ortho Tiles were collected in the study area (Fig. 3). The enhanced spectral compatibility of SuperDove with Sentinel-2 makes it particularly suitable for multi-sensor fusion [10].

Only 2022 cloud-free images over the study area were retained, and autumn imagery was excluded to avoid disturbances caused by urban tree pruning. Such interventions, which vary across species and locations, can alter canopy structure and spectral signals, potentially biasing species classification.

#### 4.1.3 Images co-registration

Systematic offsets and acquisition artifacts on acquisition dates can cause geometric and spectral inconsistencies in satellite imagery. To address this, a coregistration step is essential—especially when working with multi-sensor time series in urban environments [11]. Sentinel-2 images were co-registered using the *CO-REGIS* algorithm [12]. The processing chain allowing to align each band independently to a single spring-date reference was carried out on the EOST-A2S (Application Satellite Survey) HPC facility of the University of Strasbourg, part of the Data Terra Research Infrastructure. Similarly, PlanetScope images were co-registered using the *AROSICS* Python package [13], which performs subpixel alignment through a multistage workflow and minimizes false positives. *AROSICS* was also used to align the PlanetScope reference image with the Sentinel-2 spatial grid, ensuring full consistency between sensors.

#### 4.1.4 Polygon-based value extraction

For both sensors, the reflectance values were extracted as the median within a 1-meter buffer around each point of the city tree dataset, ensuring consistent sampling of high-quality pixels over time. This approach aims to capture the representative spectral signal of the pixel directly beneath each tree while also accounting for potential geolocation uncertainties and edge effects at pixel boundaries. The extracted time series match the corresponding attributes—locations, IDs, and species labels—of this original tree city database.

### 4.2 Deep learning models

We evaluated three deep learning configurations based on InceptionTime architecture [8] to classify urban tree species with different satellite image time series (SITS):

• InceptionTime-S2: a single-branch model trained on Sentinel-2 (S2) SITS;
• InceptionTime-PS: a single-branch model trained on PlanetScope (PS) SITS;
• Dual-InceptionTime: a dual-branch architecture jointly trained on both S2 and PS SITS with feature-level fusion.

InceptionTime is specifically designed for multivariate time series classification and includes several enhancements such as inception modules with multi-scale convolutions, residual connections, and bottleneck layers, enabling it to effectively capture both short- and long-term temporal dependencies. Each model processes the temporal-spectral signatures of trees to capture phenological variations specific to tree species.

For the dual-source configuration, we adopted a feature-level fusion approach by concatenating the latent representations extracted by each branch before the final dense classification layer. This enables the model to jointly exploit the complementary characteristics of both sources: S2 provides richer spectral information (10–20m spatial resolution, 5-day revisit, 10 bands), while PS offers finer spatial and temporal granularity (3.125m spatial resolution, near-daily revisit, 4 bands). This fusion strategy was chosen because it is well-suited to deep learning architectures and enables the model to learn joint representations across sources [14].

#### 4.2.1 Implementation details

The model was trained for 30 epochs using the Adam optimizer with an initial learning rate of $10^{-3}$ and a learning rate decay strategy to improve convergence. To account for the class imbalance typical in urban tree inventories, we used a weighted categorical cross-entropy loss. The weight assigned to each class was calculated as the inverse of its relative frequency in the dataset, following a common practice in multi-class remote sensing classification [15].

The dataset was divided into training (52.5%), validation (17.5%) and test sets (30%). The test set was intentionally oversized to support a downstream error pattern analysis. Each tree in the dataset includes a *set* field indicating its assignment. A 5-fold cross-validation scheme was implemented to ensure robustness in the evaluation.

Data were normalized using the min/max method for each urban tree species [16]:

$$x_{i,c,t}^{norm}=\frac{x_{i,c,t}-min_{j\epsilon D_{s}}x_{j,c,.}}{max_{j\epsilon D_{s}}-\;min_{j\epsilon D_{s}}x_{j,c,.}}$$

Where:

• $x_{i,c,t}$ is the raw value of sample i, for channel c (e.g., a spectral band or index), at time t;
• $x_{i,c,t}^{norm}$is the normalized value;
• $D_{s}$ denotes the set of all samples belonging to species s, i.e., the same species as sample i;
• $x_{j,c,.}$ refers to all values of channel c, across all time steps for sample j;
• $min_{j\epsilon D_{s}}x_{j,\;c,\;.}$ and $max_{j\epsilon D_{s}}$ are respectively the minimum and maximum values observed for species s and channel c, across all time steps and all samples j in $D_{s}$

This normalization scales the input values to the [O;1] range, separately for each species and spectral channel, without altering the temporal structure of the original time series.

To mitigate risks of overfitting due to spatial autocorrelation, especially in structured urban environments, particular attention was paid to the independence of spatial units across splits. Trees belonging to the same planting formation (e.g., trees aligned along a street with similar species and planting dates) or located within 50 meters of each other were not assigned to different subsets. These formations were identified using the *Patrimoine arboré 2022* municipal database.

### 4.3 Baseline results

#### 4.3.1 Urban tree species classification outputs

We compared three deep learning configurations for urban tree species classification based on SITS, using a fixed taxonomy of 20 species. The results are reported in Table 1 as mean overall accuracies across 5-fold cross-validation.

**Table 1:** Runs by SITS input and overall accuracy.

Run encoding	S2 SITS	PS SITS	Number of species	Accuracy*
Dual-InceptionTime	✓	✓	20	0.656 ± 0.005
InceptionTime-S2	✓		20	0.603 ± 0.003
InceptionTime-PS		✓	20	0.615 ± 0.004

⁎ Across 5-fold cross-validation.

The Dual-InceptionTime model, which integrates both S2 and PS time series through a feature-level fusion strategy, achieved the best performance, with an average accuracy of 0.656 ± 0.005. In comparison, single-source models obtained slightly lower results: InceptionTime-PS reached 0.615 ± 0.004, and InceptionTime-S2 achieved 0.603 ± 0.003.

These findings confirm the added value of multi-source fusion in urban tree species classification tasks. Despite their inherent differences, the SITS achieved improved performance through their complementarity, underscoring the benefits of integrating diverse temporal and spectral information sources.

#### 4.3.2 Latent space visualization and error analysis: t-SNE

**Fig. 4:**
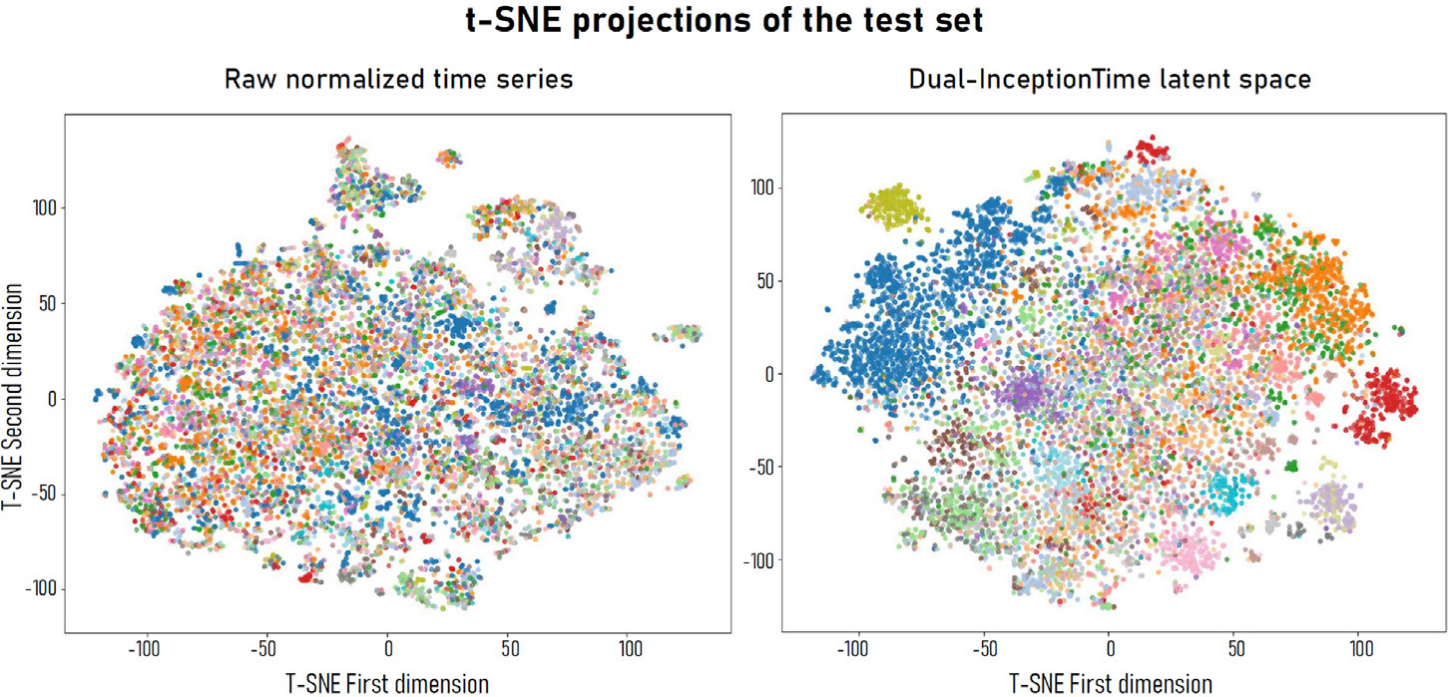
t-SNE projections of the test set: raw normalized time series (left) vs. Dual-InceptionTime latent space (right). Each dot represents a tree, colored according to its species label.

To support post hoc explainability, we analyzed the feature space learned by the Dual-InceptionTime model using t-distributed stochastic neighbor embedding (t-SNE). Fig. 4 compares the projection of the raw normalized time series (input data) with the projection of the latent representations obtained from the last layer of the dual-branch model (before classification). Although the raw data show a largely unstructured space, the learned characteristics exhibit distinct clusters, indicating the model’s ability to disentangle spectral-temporal patterns associated with tree species.

To facilitate interpretation and error analysis, we developed an interactive 3D t-SNE interface. Correct and incorrect predictions are visualized using different markers, with colors denoting tree species. For each class, we computed the centroid of the correctly classified samples using a kernel density estimation (KDE) in the t-SNE space. These centroids capture the most representative embedding for each species. Misclassified samples are then linked to their nearest class centroids using Euclidean distance, providing insight into inter-class confusion and species similarity in the learned representation space.

This approach offers an intuitive, spatially explicit way to explore model behavior and error patterns, aligning with the broader objective of model- and data-driven explainability. The interface enables users to filter species, visualize error clusters, and investigate confusion mechanisms at the latent level, complementing quantitative performance metrics. All the code is available on Github.

## Limitations

Model inference was not evaluated beyond the original dataset. Although the dataset is complete and well-structured, training a model on this data and applying it to other sensors or acquisition dates (i.e. another area) remains challenging. Differences in spectral characteristics, temporal resolution, or viewing conditions can hinder generalization and lead to decreased classification performance. More research is required to assess the transferability and robustness of the models in new urban contexts.

## Ethics Statement

This dataset does not involve human subjects, animal experiments, or sensitive data.

## CRediT Author Statement

**Clément Bressant**: Conceptualization, Methodology, Formal analysis, Software, Investigation, Data curation, Writing – original draft, Writing – review & editing. **Romain Wenger**: Writing – original draft, Writing - Review & Editing, Methodology, Software, Supervision, Validation, Funding acquisition. **David Michéa**: Resources, Data Curation. **Anne Puissant**: Writing - Review & Editing, Supervision, Funding acquisition.

## Declaration of Competing Interest

The authors declare that they have no known competing financial interests or personal relationships that could have appeared to influence the work reported in this paper.

## Data Availability

EasyData - Data TerraUrban tree species benchmark dataset for time series classification (Original data)
